# Hospital utilization rates for influenza and RSV: a novel approach and critical assessment

**DOI:** 10.1186/s12963-021-00252-5

**Published:** 2021-06-14

**Authors:** Emily K. Johnson, Dillon Sylte, Sandra S. Chaves, You Li, Cedric Mahe, Harish Nair, John Paget, Tayma van Pomeren, Ting Shi, Cecile Viboud, Spencer L. James

**Affiliations:** 1 Institute of Health Metrics and Evaluation, University of Washington, Seattle, USA; 2 Foundation for Influenza Epidemiology, Fondation de France, Paris, France; 3 Vaccine Epidemiology and Modeling Department, Sanofi Pasteur, Lyon, France; 4 Centre for Global Health, Usher Institute, University of Edinburgh, Edinburgh, UK; 5 Netherlands Institute for Health Services Research (Nivel), Utrecht, Netherlands; 6 Fogarty International Center, National Institutes of Health, Bethesda, USA

**Keywords:** Influenza, Respiratory syncytial virus, Acute lower respiratory infections, Inpatient admissions

## Abstract

**Background:**

Influenza and respiratory syncytial virus (RSV) contribute significantly to the burden of acute lower respiratory infection (ALRI) inpatient care, but heterogeneous coding practices and availability of inpatient data make it difficult to estimate global hospital utilization for either disease based on coded diagnoses alone.

**Methods:**

This study estimates rates of influenza and RSV hospitalization by calculating the proportion of ALRI due to influenza and RSV and applying this proportion to inpatient admissions with ALRI coded as primary diagnosis. Proportions of ALRI attributed to influenza and RSV were extracted from a meta-analysis of 360 total sources describing inpatient hospital admissions which were input to a Bayesian mixed effects model over age with random effects over location. Results of this model were applied to inpatient admission datasets for 44 countries to produce rates of hospital utilization for influenza and RSV respectively, and rates were compared to raw coded admissions for each disease.

**Results:**

For most age groups, these methods estimated a higher national admission rate than the rate of directly coded influenza or RSV admissions in the same inpatient sources. In many inpatient sources, International Classification of Disease (ICD) coding detail was insufficient to estimate RSV burden directly. The influenza inpatient burden estimates in older adults appear to be substantially underestimated using this method on primary diagnoses alone. Application of the mixed effects model reduced heterogeneity between countries in influenza and RSV which was biased by coding practices and between-country variation.

**Conclusions:**

This new method presents the opportunity of estimating hospital utilization rates for influenza and RSV using a wide range of clinical databases. Estimates generally seem promising for influenza and RSV associated hospitalization, but influenza estimates from primary diagnosis seem highly underestimated among older adults. Considerable heterogeneity remains between countries in ALRI coding (i.e., primary vs non-primary cause), and in the age profile of proportion positive for influenza and RSV across studies. While this analysis is interesting because of its wide data utilization and applicability in locations without laboratory-confirmed admission data, understanding the sources of variability and data quality will be essential in future applications of these methods.

**Supplementary Information:**

The online version contains supplementary material available at 10.1186/s12963-021-00252-5.

## Background

Despite the large burden of lower respiratory infections globally [1], it is difficult to estimate the proportion of the hospitalizations attributable to influenza and respiratory syncytial virus (RSV) across countries or over time. Heterogeneous coding practices in hospital records across countries limit the comparability of administrative datasets from different locations and pose a challenge to producing global hospitalization estimates using influenza and RSV-coded inpatient admissions alone. Without the addition of laboratory test result data, administrative data may not accurately estimate inpatient disease burden, further complicating efforts to model burden at the population level. Absent accurate population estimates of the burden of specific respiratory diseases, it will be challenging to conduct cross-country comparison, a hallmark of linking health policies (e.g., masking, vaccination campaigns) to outcomes.

The Burden of Influenza and RSV Disease (BIRD) project has developed an alternative method that may be useful for producing estimates of county-specific influenza and RSV burdens using administrative hospitalization data. This method generates rates of influenza and RSV-related acute lower respiratory illness (ALRI) hospitalizations across 44 countries by modeling the proportion of ALRI hospitalizations specifically attributable to RSV and influenza from literature estimates of laboratory-confirmed influenza and RSV among ALRI hospitalizations. The model can be applied to administrative data on country-specific influenza and RSV utilization. By comparing the results of the BIRD project method to those produced by raw extraction of ICD-coded RSV and influenza admission rates, we can estimate the potential under-attribution of ALRI to these specific causes.

## Methods

**Fig. 1:**
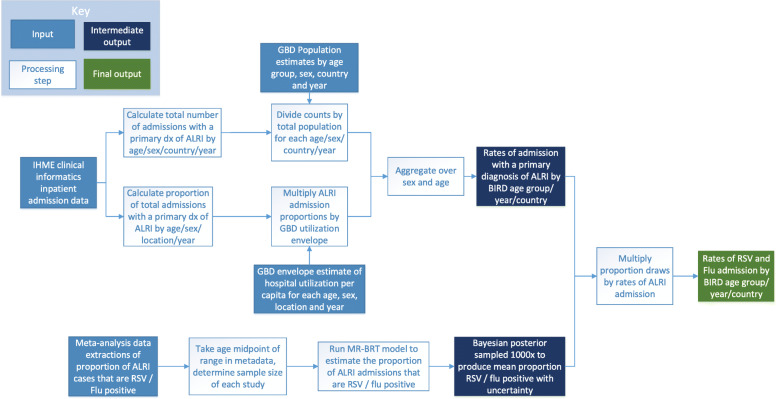
Flowchart of ALRI admission processing and meta-analysis modeling. Flowchart of data processing and analysis conducted under this study. This diagram describes processing of ALRI admissions from clinical administrative data as well as the modeling and processing performed on RSV and Influenza meta-analysis proportions

At a high level, this study estimates influenza and RSV admission rates by modeling the proportion of ALRI admissions that are due to influenza and RSV respectively, and then multiplying these proportions by ALRI admission rates from clinical administrative data. Figure 1 below is a detailed flowchart of the processing steps used in this analysis, and each step is described in further detail in the following sections.

### ALRI admissions calculation

We extracted admission counts for ALRI from 29 inpatient all-cause admission datasets covering 44 countries and containing hospitalizations spanning the years 1990 to 2017, stratified by age in years or age groups depending on the source. These datasets included approximately 43 million admissions and represent all ICD-coded inpatient admission data used in the Global Burden of Disease Study, an international collaborative study led by the Institute for Health Metrics and Evaluation (IHME) at the University of Washington and supported by over 4800 researchers in more than 140 countries [1]. Additional detail on inpatient data from IHME is listed in Additional file 1. Because only 11 of the 44 datasets utilized in this study recorded secondary diagnoses, ALRI admissions were defined as those with a primary diagnosis code listed in Table 1 below.

**Table 1:** Acute lower respiratory infection ICD codes

ICD Version	Code	Description
ICD-10	J10	Influenza due to identified seasonal influenza virus
ICD-10	J11	Influenza, virus not identified
ICD-10	J12	Viral pneumonia, not elsewhere classified
ICD-10	J13	Pneumonia due to Streptococcus pneumoniae
ICD-10	J14	Pneumonia due to Haemophilus influenzae
ICD-10	J15	Bacterial pneumonia, not elsewhere classified
ICD-10	J16	Pneumonia due to other infectious organisms, not elsewhere classified
ICD-10	J18	Pneumonia, organism unspecified
ICD-10	J20	Acute bronchitis
ICD-10	J21	Acute bronchiolitis
ICD-10	J22	Unspecified acute lower respiratory infection
ICD-9	466	Acute bronchitis and bronchiolitis
ICD-9	480	Viral pneumonia
ICD-9	481	Pneumococcal pneumonia
ICD-9	482	Other bacterial pneumonia
ICD-9	483	Pneumonia due to other specified organism
ICD-9	484	Pneumonia in infectious diseases classified elsewhere
ICD-9	485	Bronchopneumonia, organism unspecified
ICD-9	486	Pneumonia, organism unspecified
ICD-9	487	Influenza
ICD-9	488	Influenza due to identified avian influenza virus

ICD codes used to identify ALRI primary admissions. Note that all more detailed codes below those listed were also included

The majority of clinical datasets in this analysis contain a subset of the country’s total inpatient utilization. For these non-comprehensive clinical sources, counts of ALRI admissions by age were divided by the total number of admissions in the dataset to produce age-specific proportions of inpatient utilization that have a primary ALRI diagnosis. This proportion is multiplied by IHME’s total inpatient utilization envelope to approximate a comprehensive rate of ALRI utilization by age and country. The envelope is produced using a spatio-temporal Gaussian process regression that smooths over geographic distance and year of hospitalization and that models admission rate per capita by age using IHME’s healthcare access quality indicator, supply of inpatient hospital beds, and all-cause mortality as predictive covariates. More detail on the envelope estimation process, covariates used in the model, and results can be found in related Global Burden of Disease (GBD) publications [1].

The UK Hospital Episode Statistics dataset [2] and Healthcare Cost and Utilization Project National Inpatient Sample (HCUP NIS) [3] are considered comprehensive datasets and the scaling described above was not applied to these sources. Instead, counts of admissions with a primary ALRI diagnosis in these sources were divided by the total population of that country to produce rates of ALRI admission by BIRD age group and year. Population estimates are produced as part of IHME’s GBD study and detailed information on the methods to produce these estimates are available in related publications [1].

Most clinical administrative data is provided in age in years or occasionally in various aggregated age bins. The age groupings used for the BIRD analysis were at a higher level of aggregation than the majority of administrative sources used. Therefore, the final step in ALRI admission processing was to aggregate rate-space estimates to the BIRD analysis age groups, by summing both the numerator and denominator so that the rates of ALRI utilization are binned appropriately to match the rest of the analysis.

While many of the data sources used in this analysis are also used in creating annual GBD estimates, there were some differences in data processing methods between the two projects that led to different estimates of rates of ALRI. GBD analysis adjusts inpatient data to account for readmissions, potential missingness of secondary inpatient diagnoses, unavailable outpatient data, and healthcare access and quality for every location. It aggregates inpatient data with claims and outpatient data to produce estimates of individuals who received any care for an ALRI diagnosis. Because this study was primarily focused on inpatient diagnoses of influenza or RSV, these additional corrections were not applied.

### Influenza and RSV proportion estimation

Influenza and RSV admission rates were estimated by modeling the proportion of admissions for ALRI that were attributable to each cause respectively, and then estimating the proportion of total ALRI hospitalizations represented by these diseases, stratified by age, year, and country. The meta-analysis for this model included 156 independent studies on influenza-associated hospitalization rates covering 46 countries with data between 1979 and 2015 for influenza [4–159], and 204 studies on RSV admission rates covering 56 countries with data between 1982 and 2017 [4, 19, 73, 107, 133, 146, 160–356]. Sample size of the study, age range, and location in study cohort, total admissions for ALRI, and admissions for influenza and RSV respectively were extracted from each study. The proportion of ALRI admissions due to influenza and RSV were calculated for each location, age, and year present in the input study data.

A Bayesian regularized trimmed meta-regression (MR-BRT) model was generated using ALRI admission meta-analysis data to produce estimates of the proportion of ALRI admissions due to each cause while accounting for within-study heterogeneity by age and location as well as error and bias between sources. Within the MR-BRT framework, the trend over age was modeled as a cubic spline with linear tails on the youngest and oldest age groups and an uninformative Gaussian prior. Linear tails on the age ends were used to smooth behavior of the age pattern at the poles in cases of sparse data, which can be highly unstable in MR-BRT modeling.

Location was used as a covariate at the IHME Global Burden of Disease’s super-region and regional levels, to account for potential geographic variation while informing estimations for locations with sparse data by the trend of those with a larger input evidence base. Region was used as a proxy for country-level heterogeneity in order to produce estimates where meta-analysis data was available and admissions data was not or vice versa. IHME’s regional categorization by country is available in related literature. Both region and super-region were modeled as a fixed effect with an uninformative Gaussian prior on each. The hierarchical structure of the super-regional and regional models results in child models that follow the same age trend as those of the parents.

The equation for the influenza and RSV MR-BRT models is shown in Eq. 1 below. Detail on the assumptions made by the mixed effects framework, the use of cubic splines on fixed effects, and estimation of the posterior using maximum likelihood estimation are available in related literature [357]. The MR-BRT framework is an R wrapper for the open source mixed effects LimeTr package, which could be used to replicate the modeling methods described here [358].

1 $$ \ln \left({p}_{\left( flu\ \right| RSV\ \Big),i,j}\right)=\mathrm{spline}\left({\mathrm{age}}_{i,j}{\beta}_1\right)+\ln \left({\mathrm{region}}_{i,j}{\beta}_2\right)+\ln \left(\mathrm{super}\ {\mathrm{region}}_{i,j}{\beta}_3\right)+{Z}_i{u}_{i,j}+{\epsilon}_{ij} $$

Where *p*_(*flu* |*RSV*), *i*, *j*_ is the proportion of ALRI admissions that are positive for flu or RSV in observation *i* for study *j*, age_*i*, *j*_ is computed using a spline based matrix for age midpoint, region_*i*, *j*_ and super region_*i*, *j*_ are the fixed effects on GBD region and super region, *Z*_*i*_is a linear map, *u*_*i*, *j*_are the random effects from meta-analysis study *j* at observation *i*, and *ϵ*_*ij*_ are measurement errors with a specified covariance.

A hierarchical method was chosen a priori for this analysis as it allowed us to produce estimates for locations with little or no meta-analysis data while still accounting for location-specific randomness in meta-analysis estimates. In the final results of this analysis, location-level estimates maintain age heterogeneity based on the differences of age patterns for ALRI admission rates by each location.

Bootstrapping was performed by taking 1000 samples on the posterior of the MR-BRT model, and uncertainty from the samples was propagated through the remainder of the estimation process as 95% credible intervals.

### Final admission rate estimation

Admission counts and rates for influenza and RSV were calculated by multiplying the proportions from the influenza and RSV mixed effects attribution models to annual ALRI admission count estimates by age group and location. Seasonality was excluded from the scope of this analysis because seasonal information was not consistently available in influenza and RSV meta-analysis literature. Each location with clinical data received the attribution model fit for the corresponding GBD region, unless no input data for the model existed, in which case an average of the models within the GBD super-region was used. Uncertainty was quantified using the upper and lower uncertainty interval from the fit of the mixed effects model. Due to meta-analysis data sparsity in older ages for the RSV attribution mixed effects model, admission rates and counts for RSV were only calculated for children under five.

Influenza and RSV-coded primary admissions were extracted from a subset of clinical administrative datasets as illustrative scenarios in order to compare results of the BIRD analysis to direct ICD extraction with no adjustments. ICD codes used for this comparison can be found in Additional file 2. All locations used to illustrate the comparison contained at least 4-digit ICD detail, which was required to identify primary admissions for RSV.

To assess the limitation of using primary diagnosis alone for ALRI admissions, we extracted non-primary diagnosis detail from the HCUP NIS data which was used to produce US estimates [3]. Diagnosis levels available in HCUP NIS vary by state, but all available diagnosis detail up to the 30^th^ inpatient diagnosis was included for this analysis. We compared primary and non-primary utilization for the year 2012 from this dataset, and applied influenza-attributable proportion estimates to the complete dataset in order to generate a comparison of influenza rates that include non-primary hospitalizations. We focused specifically on influenza for this sub analysis because of the substantial ALRI utilization as non-primary diagnosis in older ages, as there may be competing complications that would end up coded as primary discharge diagnosis in this population [359–362].

## Results

**Fig. 2:**
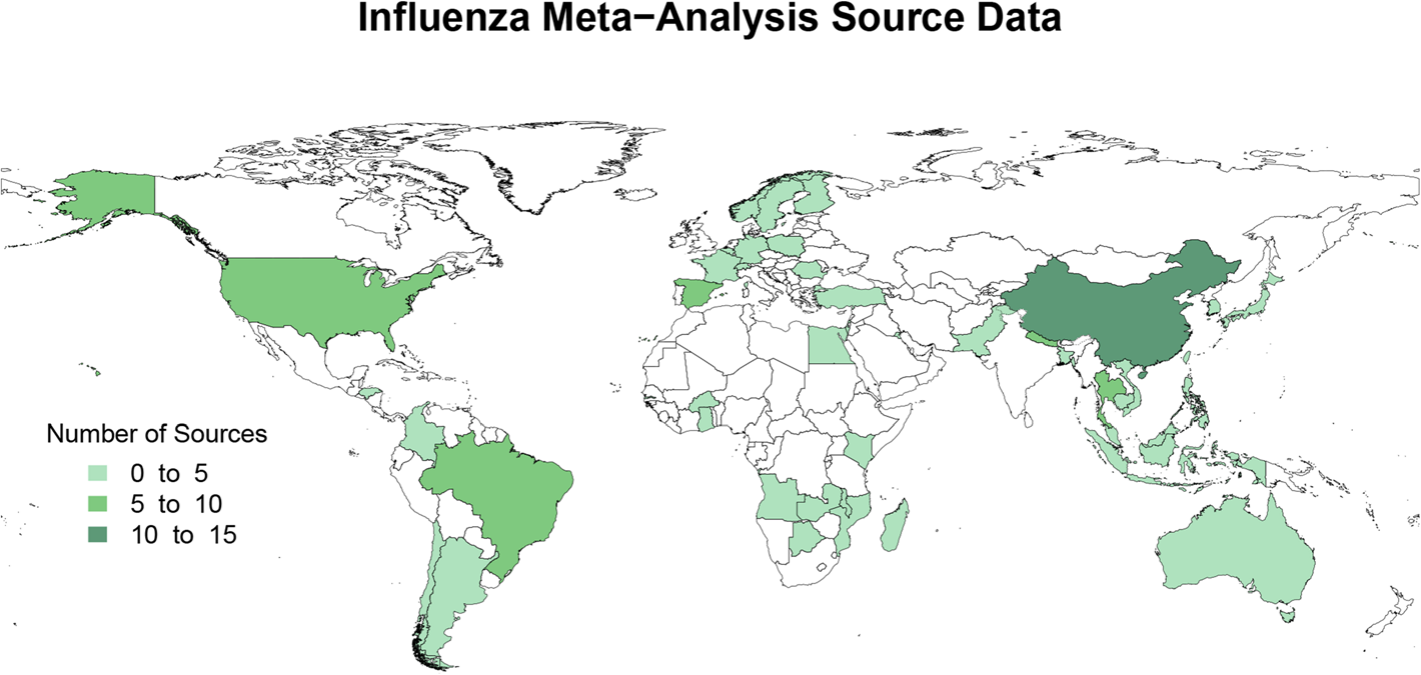
Map of influenza meta-analysis source data. Influenza meta-analysis data availability by country

**Fig. 3:**
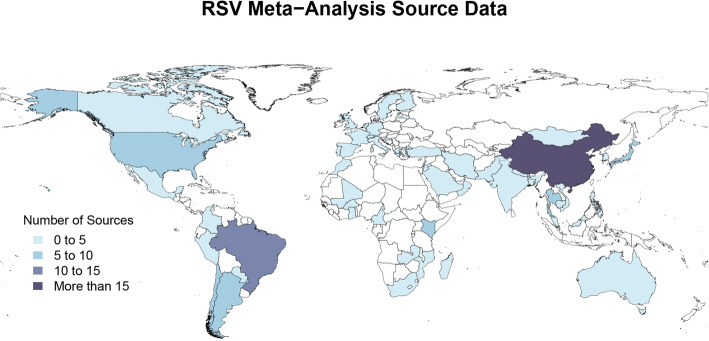
Map of RSV meta-analysis source data. RSV meta-analysis data availability by country

Figures 2 and 3 represent the number of sources of meta-analysis data for the proportion of ALRI admissions attributable to influenza and RSV, respectively. Meta-analysis sources varied in their age ranges and granularity, sample size, and the time range over which studies were conducted. All meta-analysis sources were used to inform the meta-regression analyses as described above.

Metadata about each of IHME’s inpatient data sources is available in Additional file 1. Only the inpatient sources that were ICD-9 or ICD-10 coded were used in this analysis. While all sources listed had sufficient ICD detail to extract ALRI utilization rates, not all locations with inpatient admission data have at least 4-digit ICD coding which is required to identify RSV cases by ICD diagnosis alone (see Additional file 2 for the list of 4-digit RSV codes).

**Fig. 4:**
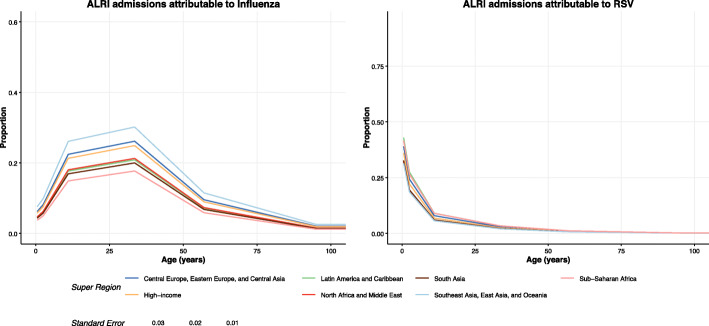
Proportion of ALRI admissions attributable to influenza and RSV. Influenza and RSV proportion models and meta-analysis input data for all IHME super-regions. Data point and model line colors reflect the GBD super region. Size of data points is scaled by the standard error of each datum

Figure 4 shows the proportion of ALRI admissions attributable to influenza and RSV at the super-regional level. Due to limited meta-data availability in older ages for RSV as seen in the figure, admission rates for RSV were only estimated for the under 1 and 1 to 4 year age groups. Data for selected regions are tabulated in Table 2 below.

**Table 2:** Proportion influenza and RSV positive by GBD super-region

GBD super region	Age	Influenza positive proportion of ALRI admissions (95% UI)	RSV positive proportion of ALRI admissions (95% UI)
Central Europe, Eastern Europe, and Central Asia	< 1 year	0.06 (0.01–0.25)	0.39 (0.14–0.72)
Central Europe, Eastern Europe, and Central Asia	1 to 4	0.08 (0.01–0.31)	0.24 (0.07–0.55)
Central Europe, Eastern Europe, and Central Asia	5 to 17	0.22 (0.03–0.63)	0.08 (0.02–0.23)
Central Europe, Eastern Europe, and Central Asia	18 to 49	0.26 (0.04–0.68)	0.03 (0.01–0.1)
Central Europe, Eastern Europe, and Central Asia	50 to 64	0.1 (0.01–0.35)	0.01 (0–0.03)
Central Europe, Eastern Europe, and Central Asia	65 plus	0.02 (0–0.09)	0 (0–0.01)
High-income	< 1 year	0.06 (0.01–0.23)	0.36 (0.12–0.68)
High-income	1 to 4	0.08 (0.01–0.29)	0.22 (0.06–0.51)
High-income	5 to 17	0.21 (0.03–0.62)	0.07 (0.02–0.2)
High-income	18 to 49	0.25 (0.04–0.67)	0.03 (0.01–0.08)
High-income	50 to 64	0.09 (0.01–0.34)	0.01 (0–0.03)
High-income	65 plus	0.02 (0–0.08)	0 (0–0.01)
Latin America and Caribbean	< 1 year	0.05 (0–0.19)	0.43 (0.16–0.76)
Latin America and Caribbean	1 to 4	0.06 (0.01–0.24)	0.27 (0.08–0.6)
Latin America and Caribbean	5 to 17	0.18 (0.02–0.56)	0.09 (0.02–0.26)
Latin America and Caribbean	18 to 49	0.21 (0.03–0.62)	0.03 (0.01–0.11)
Latin America and Caribbean	50 to 64	0.07 (0.01–0.29)	0.01 (0–0.04)
Latin America and Caribbean	65 plus	0.01 (0–0.07)	0 (0–0.01)
North Africa and Middle East	< 1 year	0.05 (0–0.19)	0.32 (0.1–0.66)
North Africa and Middle East	1 to 4	0.06 (0.01–0.25)	0.19 (0.05–0.48)
North Africa and Middle East	5 to 17	0.18 (0.02–0.56)	0.06 (0.01–0.18)
North Africa and Middle East	18 to 49	0.21 (0.03–0.61)	0.02 (0–0.07)
North Africa and Middle East	50 to 64	0.07 (0.01–0.29)	0.01 (0–0.03)
North Africa and Middle East	65 plus	0.02 (0–0.07)	0 (0–0)
South Asia	< 1 year	0.04 (0–0.18)	0.33 (0.1–0.67)
South Asia	1 to 4	0.06 (0.01–0.23)	0.19 (0.05–0.49)
South Asia	5 to 17	0.17 (0.02–0.54)	0.06 (0.01–0.18)
South Asia	18 to 49	0.2 (0.03–0.59)	0.02 (0–0.07)
South Asia	50 to 64	0.07 (0.01–0.27)	0.01 (0–0.03)
South Asia	65 plus	0.01 (0–0.06)	0 (0–0)
Southeast Asia, East Asia, and Oceania	< 1 year	0.08 (0.01–0.29)	0.31 (0.1–0.64)
Southeast Asia, East Asia, and Oceania	1 to 4	0.1 (0.01–0.36)	0.19 (0.05–0.46)
Southeast Asia, East Asia, and Oceania	5 to 17	0.26 (0.04–0.68)	0.06 (0.01–0.17)
Southeast Asia, East Asia, and Oceania	18 to 49	0.3 (0.05–0.73)	0.02 (0–0.07)
Southeast Asia, East Asia, and Oceania	50 to 64	0.11 (0.01–0.41)	0.01 (0–0.02)
Southeast Asia, East Asia, and Oceania	65 plus	0.03 (0–0.11)	0 (0–0)
Sub-Saharan Africa	< 1 year	0.04 (0–0.16)	0.42 (0.15–0.75)
Sub-Saharan Africa	1 to 4	0.05 (0–0.21)	0.27 (0.08–0.59)
Sub-Saharan Africa	5 to 17	0.15 (0.02–0.49)	0.09 (0.02–0.26)
Sub-Saharan Africa	18 to 49	0.18 (0.02–0.55)	0.03 (0.01–0.11)
Sub-Saharan Africa	50 to 64	0.06 (0.01–0.24)	0.01 (0–0.04)
Sub-Saharan Africa	65 plus	0.01 (0–0.06)	0 (0–0.01)

Proportion of ALRI admissions that are influenza and RSV positive for all IHME super-regions stratified by BIRD age group

In these results, influenza represents a significant proportion of ALRI admissions in individuals aged 15 to 55 years, and a lower proportion in the oldest and youngest age groups. Conversely, RSV represents over 30% of all ALRI admissions for infants under 1 year and over 18% for infants aged 1–4, but the proportion of ALRI admissions attributable to RSV drops dramatically in age groups beyond the age of 5 years.

**Fig. 5:**
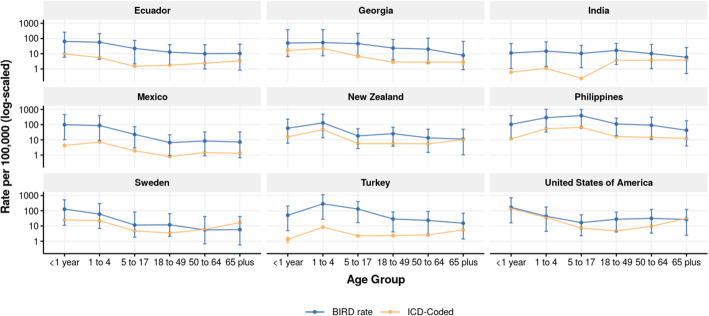
Influenza admission rate by BIRD analysis and ICD coding. Influenza admission rate per 100,000 people by age as produced by BIRD analysis (blue) and simple raw ICD code extraction (yellow). 95% CI shown for both estimates

**Fig. 6:**
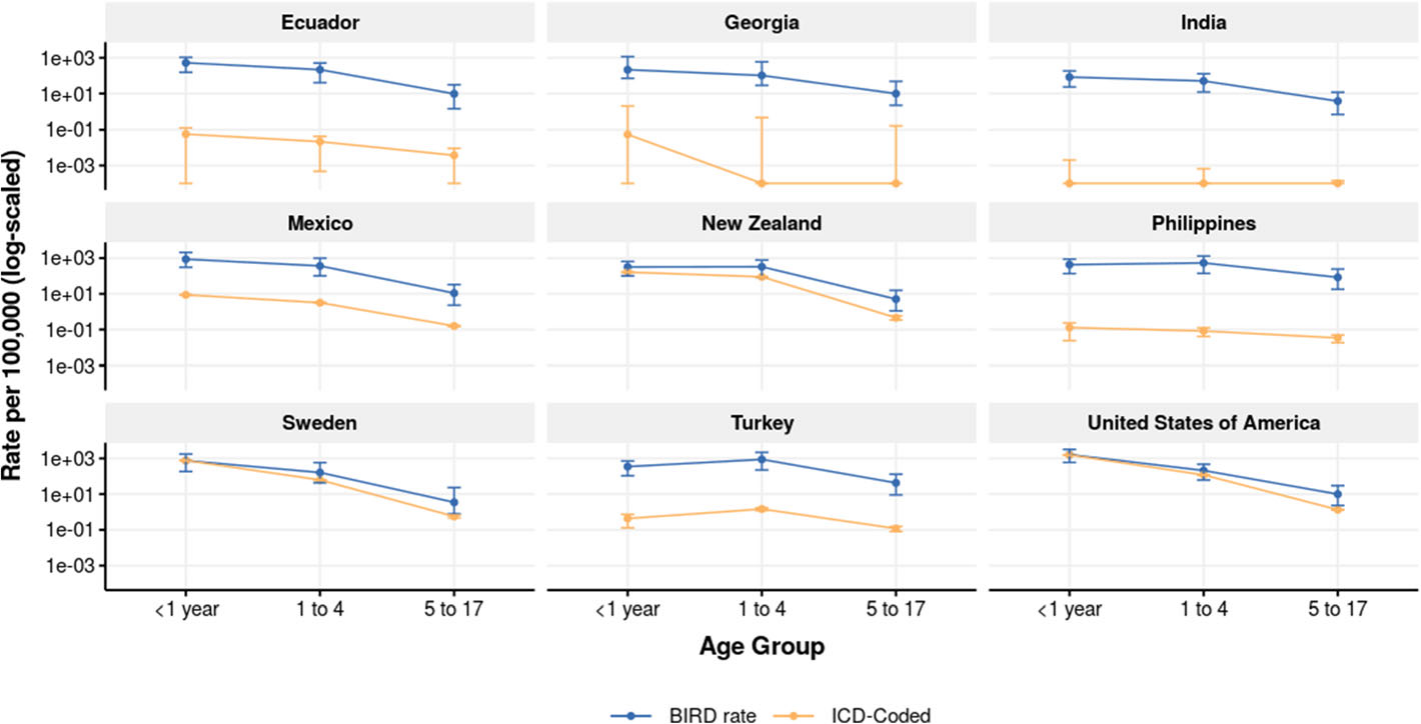
RSV admission rate by BIRD analysis and ICD coding. RSV admission rate per 100,000 by age as produced by BIRD analysis (blue) and simple raw ICD code extraction (yellow). 95% CI shown for both estimates

Comparisons of admission rates calculated through the BIRD analysis versus those coded directly with influenza and RSV ICD codes for locations with sufficient ICD granularity are shown in Figs. 5 and 6, and tabulated in Tables 3 and 4. For almost all age groups, the methods as described in this paper estimated a higher national admission rate than the rate of directly coded influenza or RSV admissions in the same inpatient sources. Many inpatient data sources used at IHME are coded only to three or four digits, in which case it is less accurate or even not possible to estimate RSV admission rates. Detail on inpatient clinical sources and ICD granularity is listed in Additional file 1, and the ICD codes used to determine influenza and RSV inpatient admissions are listed in Additional file 2. The full dataset of BIRD estimates of influenza and RSV admissions by age, year, and country are available in Additional file 3.

**Table 3:** Influenza rates by BIRD analysis and ICD code extraction for select locations

Country	Age group	BIRD rate per 100,000 (95% UI)	ICD-coded rate per 100,000 (95% UI)
Ecuador	< 1 year	64.5 (6–260.9)	9.8 (9.1–10.6)
Ecuador	1 to 4	56.5 (4.3–210)	5.5 (5.2–5.8)
Ecuador	5 to 17	22.1 (2.2–75.5)	1.5 (1.4–1.6)
Ecuador	18 to 49	12.8 (1.7–39.6)	1.8 (1.7–1.8)
Ecuador	50 to 64	10 (1–39.1)	2.4 (2.2–2.6)
Ecuador	65 plus	10.5 (0.8–42.7)	3.4 (3.1–3.7)
Georgia	< 1 year	50.3 (6.5–375.9)	16.2 (9.7–22.7)
Georgia	1 to 4	54 (7.3–385.5)	22.5 (18.8–26.1)
Georgia	5 to 17	46.2 (8–219.6)	6.8 (5.6–8)
Georgia	18 to 49	23.5 (4–87.6)	2.8 (2.4–3.3)
Georgia	50 to 64	19.8 (2.4–107.2)	2.8 (2.1–3.5)
Georgia	65 plus	7.9 (0.9–64.8)	2.8 (2–3.6)
India	< 1 year	11.1 (1–46.4)	0.6 (0.6–0.7)
India	1 to 4	14.9 (1.5–59.8)	1.1 (1.1–1.1)
India	5 to 17	10.5 (1.2–35.1)	0.2 (0.2–0.2)
India	18 to 49	16.6 (2–47.7)	3.7 (3.7–3.7)
India	50 to 64	10.2 (1–40.8)	3.8 (3.7–3.8)
India	65 plus	5.9 (0.5–25.4)	3.9 (3.8–3.9)
Mexico	< 1 year	98.4 (10.3–458.1)	4.3 (4.1–4.5)
Mexico	1 to 4	87 (9.3–388.3)	7.1 (7–7.2)
Mexico	5 to 17	22.8 (3–74)	1.9 (1.9–2)
Mexico	18 to 49	6.6 (0.9–21.6)	0.8 (0.8–0.8)
Mexico	50 to 64	8.4 (0.9–33.2)	1.5 (1.4–1.5)
Mexico	65 plus	7.3 (0.7–32.7)	1.3 (1.2–1.4)
New Zealand	< 1 year	58 (6–231.5)	15.7 (13.3–18.1)
New Zealand	1 to 4	131.6 (13.6–482.9)	46.7 (44.6–48.8)
New Zealand	5 to 17	18.1 (2.7–54)	5.7 (5.3–6.1)
New Zealand	18 to 49	25.4 (3.8–66.7)	5.7 (5.5–6)
New Zealand	50 to 64	13.6 (1.5–50.1)	5.5 (5.1–5.9)
New Zealand	65 plus	11.3 (1–50.2)	10.5 (9.8–11.2)
Philippines	< 1 year	104.4 (11.4–394.8)	11.6 (10.7–12.5)
Philippines	1 to 4	288.2 (32.6–1024)	53.7 (52.6–54.7)
Philippines	5 to 17	390.7 (60.8–1007.2)	66.2 (65.5–66.8)
Philippines	18 to 49	110.6 (18.8–267.5)	16.3 (16–16.6)
Philippines	50 to 64	91.4 (10.7–310.1)	14.5 (14–15)
Philippines	65 plus	42.5 (3.9–179.1)	12.3 (11.6–13.1)
Sweden	< 1 year	128.5 (11.1–523.3)	25.2 (22.8–27.7)
Sweden	1 to 4	60.1 (6.9–294.4)	22.3 (21.2–23.5)
Sweden	5 to 17	11.6 (1.8–82.7)	4.8 (4.5–5.1)
Sweden	18 to 49	12 (2.1–63.8)	3.5 (3.3–3.6)
Sweden	50 to 64	5.5 (0.7–41.9)	5.9 (5.6–6.2)
Sweden	65 plus	5.8 (0.6–41.7)	16.9 (16.2–17.5)
Turkey	< 1 year	50.8 (4.9–205.6)	1.3 (0.8–1.8)
Turkey	1 to 4	284.7 (28–1137)	8.4 (7.8–9)
Turkey	5 to 17	130.3 (16.5–400.1)	2.2 (2.1–2.4)
Turkey	18 to 49	28.9 (4.1–83.5)	2.3 (2.2–2.4)
Turkey	50 to 64	23.2 (2.4–90.7)	2.6 (2.4–2.8)
Turkey	65 plus	15.3 (1.4–68.9)	5.6 (5.2–6.1)
United States of America	< 1 year	168.4 (16.2–714.1)	139.5 (138.4–140.7)
United States of America	1 to 4	43.4 (4.4–175.8)	36.1 (35.8–36.4)
United States of America	5 to 17	16.6 (2.3–54.5)	7.2 (7.1–7.3)
United States of America	18 to 49	27.8 (4.1–80.9)	4.8 (4.8–4.8)
United States of America	50 to 64	31.6 (3.4–122.6)	9.6 (9.5–9.6)
United States of America	65 plus	27 (2.5–122.6)	32 (31.8–32.2)

BIRD estimates of rates of influenza admission as compared to the rate from a raw ICD code extraction. BIRD and raw coded rate are produced across all years of available data for each country

**Table 4:** RSV rates by BIRD analysis and ICD code extraction for select locations

Country	Age group	BIRD Rate per 100,000 (95% UI)	ICD-coded rate per 100,000 (95% UI)
Ecuador	< 1 year	525.9 (154.2–1069.9)	0.1 (0–0.1)
Ecuador	1 to 4	216.4 (40.5–508.3)	0 (0–0)
Georgia	< 1 year	214.2 (71.2–1163.1)	0.1 (0–2)
Georgia	1 to 4	102.9 (28.6–595.6)	0 (0–0.5)
India	< 1 year	83.4 (23.3–184.1)	0 (0–0)
India	1 to 4	50.3 (12.1–127.4)	0 (0–0)
Mexico	< 1 year	865.6 (306.8–2077.7)	8.8 (8.4–9.1)
Mexico	1 to 4	364.3 (101.4–992.8)	3.2 (3.1–3.3)
New Zealand	< 1 year	318.3 (100.4–646.3)	163.3 (155.6–171)
New Zealand	1 to 4	327.7 (82.2–781.2)	89.4 (86.5–92.2)
Philippines	< 1 year	429.4 (135.6–876.1)	0.1 (0–0.2)
Philippines	1 to 4	536.1 (138.8–1306.9)	0.1 (0–0.1)
Sweden	< 1 year	750.8 (184–1744.6)	758.7 (746.1–771.3)
Sweden	1 to 4	160.8 (43.1–581.1)	62 (60.2–63.9)
Turkey	< 1 year	347.8 (107–711.8)	0.4 (0.1–0.7)
Turkey	1 to 4	875 (224.2–2177)	1.5 (1.2–1.7)
United States of America	< 1 year	1623.6 (595.2–3201.2)	1557 (1553.2–1560.8)
United States of America	1 to 4	209.2 (61.6–469.9)	118.4 (117.9–118.9)

BIRD estimates of rates of RSV admission as compared to the rate from a raw ICD code extraction. BIRD and raw coded rate are produced across all years of available data for each country

As non-primary diagnoses were not available for the majority of sources of inpatient admission data, only primary diagnosis was used to expand the number of useable sources and retain consistency across locations. We conducted a sensitivity analysis comparing the average primary and non-primary admission rates for ALRI in the USA from 2002 to 2012 to illustrate the potential impact of limiting the analysis to ALRI as primary diagnosis only.

**Fig. 7:**
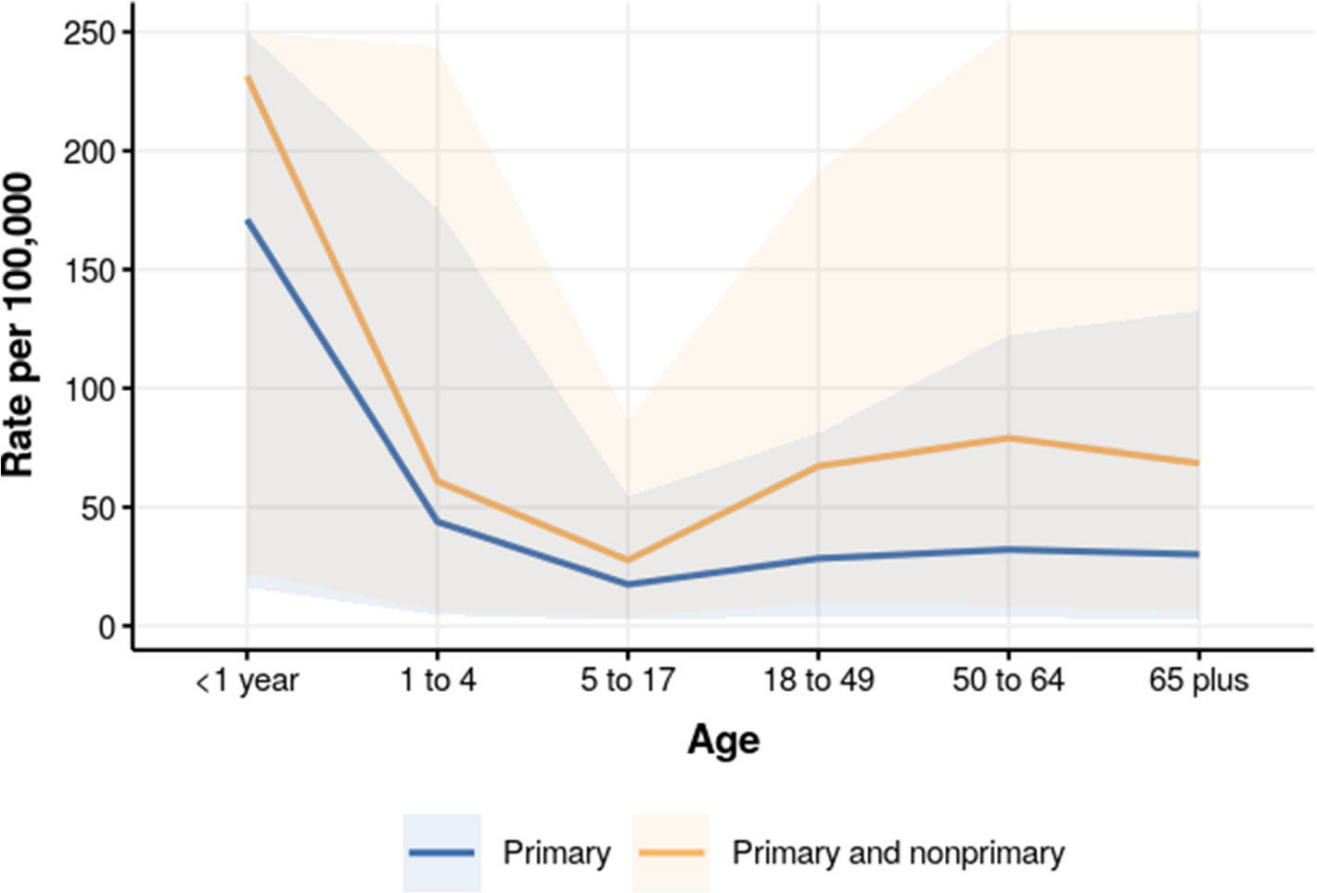
Primary versus nonprimary influenza admission rate in the USA, 2012. Influenza admission rate by diagnosis position, in US HCUP NIS data. Uncertainty is capped in order to show estimated age pattern

Influenza admission rates in the USA by primary-only diagnosis and primary and non-primary diagnosis are shown in Fig. 7. The impact of non-primary diagnoses was a 1.4-fold increase in rates estimates for children < 1 year, and nearly a 2.5-fold increase in rates estimated in the 18–49, 50 to 64, and 65 plus age groups.

## Discussion

While influenza and RSV-associated healthcare utilization is acknowledged as a global problem, gaps in quantifying the magnitude of this problem exist due to lack in representative data availability across locations that makes assessing admission rates within or across countries challenging. Traditional methods of burden estimation based on laboratory-confirmed cases are not possible in most settings because testing patients with ALRI is not routine care. This analysis utilizes clinical administrative data which is widely available across countries, and presents a means of utilization estimation that can be more robust than direct ICD extraction alone. The approach, however, has important limitations for influenza when considering older adults.

Although the true burden of RSV in children is unknown, estimates of RSV admission rates from this study are generally consistent with published literature on RSV hospital utilization in children under 5. Shi et al estimate hospital admission rates of 26.3 (22.8–30.2) per 1000 in children aged 1–5 months, 11.3 (6.1–20.9) per 1000 in children 6–11 months, and 1.4 (0.9–2.0) per 1000 in children 12–59 months old in World Bank High Income countries [363]. Reeves et al. found admission rates for RSV of 35.1 (32.9–38.9) per 1000 in children under 1 year and 5.31 (4.46–6.59) per 1000 in children age 1–4 years old in England [364]. Estimates from the BIRD analysis as shown in Table 4 are lower in high-income settings for children under 1 year of age than either study, but fall between estimates of older children as described in the literature. Further discussion and comparisons of the results of the BIRD analysis for RSV to other RSV estimation methods are available in related literature [365].

Our estimated admission rates for influenza are generally an underestimate of rates previously published, particularly in the 65+ age group [366, 367]. For the USA and Sweden at age 65+, the simple extracted ICD-coded admission rate from administrative datasets surpasses the rate produced by this study. The inclusion of non-primary diagnoses did increase estimates for influenza in the USA by more than 50%. Nonetheless, these rates are still lower than those produced by comparable studies in the oldest age group. Previous studies estimate that anywhere between 39.5 and 96.6% of all admissions across all ages for influenza have a primary diagnosis related to influenza, and the relative proportion of burden as a primary diagnosis in this analysis fall within that range [359–362]. While using only the primary diagnosis allowed us to maintain consistency with the 33 sources containing only primary diagnostic detail, future iterations of this method should consider inclusion of non-primary diagnoses for more comprehensive utilization estimation, if at the expense of geographic coverage.

Estimates of the proportion of influenza-positive adults age 65+ were also generally lower than existing literature. Jain et al. estimate that 4% adults aged 65–79 years and 5% adults 80 or older hospitalized for pneumonia in select US cities test positive for influenza [32]. Monto et al. report that 10.9% of adults aged 50 or older presenting with acute respiratory illness are influenza positive, in a study of families in Ann Arbor Michigan over 3 years [69]. Our analysis estimates 1.9% (0.02–8.4) of ALRI admissions in ages 65+ in IHME high-income settings are influenza positive cases. While the upper bound of this estimate more closely aligns with existing published literature, the proportion positive estimated from the BIRD project is low because of data sparsity in oldest ages. The age spline method used in the MR-BRT analysis depends on age midpoint of meta-analysis input data instead of accounting for an age range, which narrows the number of estimates representing older ages. Inclusion of additional meta-analysis data and incorporation of more sophisticated age range splitting could produce more robust proportion estimates in older ages.

The methodology employed by this analysis is comparable to previous burden estimates for influenza produced by IHME in the application of a proportion model to estimates of total lower respiratory infection [368]. However, estimates from the BIRD project were formed using a categorical approach that did not account for the relative risk of ALRI in cases of confirmed influenza or RSV. Instead, the proportion of ALRI hospitalizations was assumed to be a proxy of total utilization. Additionally, the BIRD analysis focuses exclusively on inpatient hospital utilization instead of incidence or mortality, which reduced the assumptions made about how trends in utilization can be extended to other metrics. Finally, the hierarchical method of modeling proportion positive by region and super-region was a novel approach used in burden analysis to allow for estimates in locations with sparser meta-analysis data to have more robust proportion estimates over age. IHME’s GBD global influenza admission rate estimates were higher than most of those predicted for countries included in BIRD analysis, at 123.8 per 100,000 (CI: 48.5–300.2) across all ages as compared to BIRD all-age rates of 29.7 per 100,000 (CI: 3.64–101.7) in the USA to 195.81 (183.88–207.74) in the Philippines.

This study met limitations that are consistent with any analysis developed from clinical administrative data. Availability of inpatient admissions data in some lower- to middle-income countries and meta-analysis data for RSV in older children and adults limited the scope of this analysis, and additional sources of both types of data would improve accuracy of estimates. Availability of inpatient data and proportion meta-analysis at a seasonal or monthly granularity would allow for more relevant analysis during peak influenza and RSV seasons. Additionally, we encountered technical limitations in handling of meta-analysis with point estimates for proportion positive spanning large age ranges, and in the assumption made that influenza and RSV proportions across countries will follow the same pattern over age. Finally, the rates estimated in this analysis represent utilization rates of influenza and RSV present in individuals who have a primary admission diagnosis of acute lower respiratory infection. Accounting for non-inpatient care including urgent or emergency departments and adjustments for non-primary diagnosis when ALRI is not the primary reason for visit would further improve the estimates produced by this analysis.

In addition to addressing the limitations described, future iterations of this methodology could be expanded to estimates of incidence or prevalence from utilization by accounting for health care access and care-seeking behavior. Furthermore, deeper investigation of goodness-of-fit of the proportion models through out of sample estimation would provide additional validation for the methods proposed here and potentially identify additional areas for refinement of the proportion models.

## Conclusions

Because of heterogeneity in coding practices between countries and limited availability of data at sufficient granularity for precise burden estimation, there are few reliable sources of influenza and RSV hospital utilization or incidence that are provided on a global scale. The application of meta-analysis for proportion positive to overall ALRI utilization is a non-traditional means of estimation that indicate promise in other applications where direct measurement of ICD diagnoses cannot provide accurate estimates of rates of disease and where surveillance data are not available. However, the method shows much uncertainty when considering influenza in older adults that could be a function of considerable heterogeneity in ALRI coding between countries (i.e., as primary vs secondary cause), and in the age profile of proportion positivity for influenza and RSV across studies. While this method is interesting because it is based on clinical administrative data that is available from many countries globally, additional refinement of admission processing methodology and inclusion of more data over ages would enable greater comparability to existing influenza and RSV utilization literature.

## Supplementary Information

**Additional file 1.** IHME Inpatient Data Metadata. Description of data: Detailed information including number of years of data, length of ICD codes, and total number of inpatient admissions for each source of clinical administrative data used in this study. All data is in the custody of the Institute of Health Metrics and Evaluation, and is available in the Global Health Data Exchange (ghdx.healthdata.org).

**Additional file 2.** Influenza and RSV ICD Codes. Description of data: The ICD-9 and ICD-10 codes used to identify influenza and RSV admissions from raw ICD extraction, to compare against the utilization rates produced by the BIRD study.

**Additional file 3.** Influenza and RSV Inpatient Admission Rates for All Country-Years of Clinical Administrative Data. Description of data: Tabulated inpatient admission rates with uncertainty for all ages and years available for each country included in the BIRD analysis. Countries where clinical administrative data from IHME was available are all included in this dataset.

## Data Availability

Inpatient admissions datasets and influenza meta-analysis data as used in this analysis are available via IHME’s Global Health Data Exchange (GHDx), http://ghdx.healthdata.org/ [2, 3, 369–390]. RSV meta-analysis data is available through the Edinburgh Datashare, https://datashare.is.ed.ac.uk/handle/10283/3611 [365].

## Abbreviations

ALRI: Acute lower respiratory infections
RSV: Respiratory syncytial virus
ICD: International Classification of Disease
BIRD: Burden of Influenza and RSV Disease
IHME: Institute for Health Metrics and Evaluation
GBD: Global Burden of Disease
MR-BRT: Bayesian regularized trimmed meta-regression
HCUP NIS: Healthcare Cost and Utilization Project National Inpatient Sample
