# Supplementary material for: Hospital utilization rates for influenza and RSV: a novel approach and critical assessment
Source: Popul Health Metr. 2021 Jun 14;19:31. doi: 10.1186/s12963-021-00252-5 (PMC8204427; doi:10.1186/s12963-021-00252-5)
Supplement: Supplementary file 1 — Additional file 1. IHME Inpatient Data Metadata. Description of data: Detailed information including number of years of data, length of ICD codes, and total number of inpatient admissions for each source of clinical administrative data used in this study. All data is in the custody of the Institute of Health Metrics and Evaluation, and is available in the Global Health Data Exchange (ghdx.healthdata.org). [file 12963_2021_252_MOESM1_ESM.docx]

## Additional File 1 IHME Inpatient Data Metadata

Additional File 1 Table 1: IHME Clinical Administrative Inpatient Metadata by Source

| **Database** | **Country** | **ICD Version** | **ICD length** | **Number of Years** | **Earliest year** | **Most recent year** | **Raw Admissions** |
| --- | --- | --- | --- | --- | --- | --- | --- |
| Austria Hospital Inpatient Discharges | Austria | Mixed | 3-4 digits | 24 | 1990 | 2014 | 61,598,000 |
| Botswana Health Management Data  System (HMDS) | Botswana | ICD-10 | 3-5 digits | 3 | 2007 | 2009 | 9,192,000 |
| Brazil Hospital Information  System (SIH) | Brazil | ICD-10 | 3-4 digits | 20 | 1997 | 2016 | 196,814,000 |
| Chile Hospital Discharge Information  System | Chile | ICD-10 | 1-6 digits | 12 | 2001 | 2012 | 22,758,000 |
| Ecuador Hospital Inpatient Discharges | Ecuador | Mixed | 3-4 digits | 18 | 1997 | 2014 | 8,714,000 |
| European Hospital Morbidity DataBase | Belgium | ICD-9 | 3-4 digits | 5 | 2003 | 2009 | 459,000 |
| European Hospital Morbidity DataBase | Croatia | ICD-9 | 3-4 digits | 12 | 2002 | 2013 | 4,589,000 |
| European Hospital Morbidity DataBase | Cyprus | ICD-9 | 3-4 digits | 8 | 2004 | 2012 | 3,759,000 |
| European Hospital Morbidity DataBase | Czech Republic | ICD-9 | 3-4 digits | 2 | 2011 | 2012 | 15,344,000 |
| European Hospital Morbidity DataBase | Denmark | ICD-9 | 3-4 digits | 4 | 2003 | 2006 | 11,131,000 |
| European Hospital Morbidity DataBase | Finland | ICD-9 | 3-4 digits | 10 | 2001 | 2012 | 4,400,000 |
| European Hospital Morbidity DataBase | Iceland | ICD-9 | 3-4 digits | 1 | 2009 | 2009 | 18,232,000 |
| European Hospital Morbidity DataBase | Latvia | ICD-9 | 3-4 digits | 9 | 2004 | 2012 | 45,000 |
| European Hospital Morbidity DataBase | Lithuania | ICD-9 | 3-4 digits | 12 | 2001 | 2012 | 421,000 |
| European Hospital Morbidity DataBase | Luxembourg | ICD-9 | 3-4 digits | 11 | 2002 | 2012 | 19,000 |
| European Hospital Morbidity DataBase | Malta | ICD-9 | 3-4 digits | 7 | 2006 | 2012 | 148,000 |
| European Hospital Morbidity DataBase | Poland | ICD-9 | 3-4 digits | 10 | 2003 | 2012 | 60,023,000 |
| European Hospital Morbidity DataBase | Romania | ICD-9 | 3-4 digits | 1 | 2012 | 2012 | 7,012,000 |
| European Hospital Morbidity DataBase | Serbia | ICD-9 | 3-4 digits | 1 | 2012 | 2012 | 81,000 |
| European Hospital Morbidity DataBase | Slovakia | ICD-9 | 3-4 digits | 10 | 2002 | 2011 | 380,000 |
| European Hospital Morbidity DataBase | Slovenia | ICD-9 | 3-4 digits | 7 | 2004 | 2012 | 10,000 |
| European Hospital Morbidity DataBase | Switzerland | ICD-9 | 3-4 digits | 11 | 2002 | 2012 | 3,817,000 |
| Georgia Hospital Data | Georgia | ICD-10 | 3-4 digits | 3 | 2013 | 2016 | 9,173,000 |
| Germany Hospital Statistics Reporting  System | Germany | ICD-10 | 3-4 digits | 1 | 2009 | 2009 | 833,000 |
| Health Care Utilization Project National  Inpatient Sample | United States | ICD-9 | 3-5 digits | 7 | 2002 | 2012 | 333,000 |
| India - Mysore JSS Hospital | India | ICD-10 | 3-4 digits | 4 | 2014 | 2017 | 23,328,000 |
| India - Shillong Nazareth Hospital  Inpatient Discharges | India | ICD-10 | 1-6 digits | 1 | 2014 | 2014 | 908,000 |
| Iran Hospital Data | Iran | ICD-10 | 3 digits | 10 | 2001 | 2010 | 9,837,000 |
| Italy - Hospital Inpatient Discharges | Italy | ICD-9 | 3-5 digits | 12 | 2005 | 2016 | 4,367,000 |
| Japan Diagnosis Procedure  Combination Database 2015 | Japan | ICD-10 | 3-5 digits | 5 | 2010 | 2015 | 11,887,000 |
| Jordan Al-Bashir Hospital Discharges  2016 | Jordan | ICD-10 | 2-3 digits | 1 | 2016 | 2016 | 57,246,000 |
| Kenya National Inpatient Morbidity  and Mortality Statistics | Kenya | ICD-10 | 3-4 digits | 1 | 1999 | 1999 | 171,000 |
| Kyrgyzstan - Bishkek Clinical-Related  Groups Hospital Claims | Kyrgyzstan | ICD-10 | 3-4 digits | 1 | 2012 | 2012 | 51,000 |
| Mexico Automated Hospital Discharge  System (SAEH) | Mexico | ICD-10 | 3-4 digits | 13 | 2000 | 2012 | 4,431,000 |
| Nepal Hospital Inpatient Discharges | Nepal | ICD-10 | 3-6 digits | 4 | 2010 | 2015 | 978,000 |
| New Zealand National Minimum  Dataset | New Zealand | Mixed | 3-5 digits | 17 | 2000 | 2016 | 10,105,000 |
| Norway Patient Register | Norway | ICD-10 | 3-4 digits | 5 | 2008 | 2012 | 2,562,000 |
| Portugal Hospital Inpatient Discharges | Portugal | ICD-9 | 3-5 digits | 1 | 2015 | 2015 | 14,922,000 |
| Qatar - Annual Inpatients Discharge  Abstract: Hamad General Hospital | Qatar | ICD-9 | 3 digits | 2 | 2002 | 2003 | 15,210,000 |
| St. Johns Medical Hospital Inpatient  Data | India | ICD-10 | 3-4 digits | 1 | 2017 | 2017 | 110,437,000 |
| Sweden National Patient Register | Sweden | ICD-10 | 3-4 digits | 17 | 1998 | 2016 | 622,674,000 |
| Turkey Diagnosis-Related Group  Hospital Inpatient Database | Turkey | ICD-10 | 3-5 digits | 2 | 2011 | 2012 | 273,000 |
| United Kingdom - England Hospital  Episode Statistics | United Kingdom | ICD-10 | 3-5 digits | 15 | 2001 | 2015 | 119,000 |
