# Supplementary material for: Hospital utilization rates for influenza and RSV: a novel approach and critical assessment
Source: Popul Health Metr. 2021 Jun 14;19:31. doi: 10.1186/s12963-021-00252-5 (PMC8204427; doi:10.1186/s12963-021-00252-5)
Supplement: Supplementary file 2 — Additional file 2. Influenza and RSV ICD Codes. Description of data: The ICD-9 and ICD-10 codes used to identify influenza and RSV admissions from raw ICD extraction, to compare against the utilization rates produced by the BIRD study. [file 12963_2021_252_MOESM2_ESM.docx]

## Additional File 2 Influenza and RSV ICD Codes

**Additional File 2 Table 1: ICD 10 codes used for Influenza to compare against BIRD analysis rates**

| **Code** | **Description** |
| --- | --- |
| J09 | Influenza due to certain identified influenza viruses |
| J10 | Influenza due to other identified influenza virus |
| J10.0 | Influenza due to other identified influenza virus with pneumonia |
| J10.00 | Influenza due to other identified influenza virus with unspecified type of pneumonia |
| J10.01 | Influenza due to other identified influenza virus with the same other identified influenza virus pneumonia |
| J10.08 | Influenza due to other identified influenza virus with other specified pneumonia |
| J10.1 | Influenza due to other identified influenza virus with other respiratory manifestations |
| J10.2 | Influenza due to other identified influenza virus with gastrointestinal manifestations |
| J10.8 | Influenza due to other identified influenza virus with other manifestations |
| J10.81 | Influenza due to other identified influenza virus with encephalopathy |
| J10.82 | Influenza due to other identified influenza virus with myocarditis |
| J10.83 | Influenza due to other identified influenza virus with otitis media |
| J10.89 | Influenza due to other identified influenza virus with other manifestations |
| J11 | Influenza due to unidentified influenza virus |
| J11.0 | Influenza due to unidentified influenza virus with pneumonia |
| J11.00 | Influenza due to unidentified influenza virus with unspecified type of pneumonia |
| J11.08 | Influenza due to unidentified influenza virus with specified pneumonia |
| J11.1 | Influenza due to unidentified influenza virus with other respiratory manifestations |
| J11.2 | Influenza due to unidentified influenza virus with gastrointestinal manifestations |
| J11.8 | Influenza due to unidentified influenza virus with other manifestations |
| J11.81 | Influenza due to unidentified influenza virus with encephalopathy |
| J11.82 | Influenza due to unidentified influenza virus with myocarditis |
| J11.83 | Influenza due to unidentified influenza virus with otitis media |
| J11.89 | Influenza due to unidentified influenza virus with other manifestations |

**Additional File 2 Table 2: ICD 9 codes used for Influenza to compare against BIRD analysis rates**

| **Code** | **Description** |
| --- | --- |
| 480.2 | Pneumonia due to parainfluenza virus |
| 480.3 | Pneumonia due to SARS-associated coronavirus |
| 487 | Influenza |
| 487.0 | Influenza with pneumonia |
| 487.1 | Influenza with other respiratory manifestations |
| 487.8 | Influenza with other manifestations |
| 488 | Influenza due to certain identified influenza viruses |
| 488.0 | Influenza due to identified avian influenza virus |
| 488.01 | Influenza due to identified avian influenza virus with pneumonia |
| 488.02 | Influenza due to identified avian influenza virus with other respiratory manifestations |
| 488.09 | Influenza due to identified avian influenza virus with other manifestations |
| 488.1 | Influenza due to identified 2009 H1N1 influenza virus |
| 488.11 | Influenza due to identified 2009 H1N1 influenza virus with pneumonia |
| 488.12 | Influenza due to identified 2009 H1N1 influenza virus with other respiratory manifestations |
| 488.19 | Influenza due to identified 2009 H1N1 influenza virus with other manifestations |
| 488.8 | Influenza due to novel influenza A |
| 488.81 | Influenza due to identified novel influenza A virus with pneumonia |
| 488.82 | Influenza due to identified novel influenza A virus with other respiratory manifestations |
| 488.89 | Influenza due to identified novel influenza A virus with other manifestations |

**Additional File 1 Table 3: ICD 10 codes used for RSV to compare against BIRD analysis rates**

| Code | Description |
| --- | --- |
| B97.4 | Respiratory syncytial virus as the cause of diseases classified elsewhere |
| J21.0 | Acute bronchiolitis due to respiratory syncytial virus |
| J20.5 | Acute bronchitis due to respiratory syncytial virus |
| J12.1 | Respiratory syncytial virus pneumonia |

**Additional File 1 Table 4: ICD 9 codes used for RSV to compare against BIRD analysis rates**

| **Code** | **Description** |
| --- | --- |
| 466.11 | Acute bronchiolitis due to respiratory syncytial virus (RSV) |
| 480.1 | Pneumonia due to respiratory syncytial virus |
| 079.6 | Respiratory synctial virus |
